# Allelic sequence heterozygosity in single *Giardia* parasites

**DOI:** 10.1186/1471-2180-12-65

**Published:** 2012-05-03

**Authors:** Johan Ankarklev, Staffan G Svärd, Marianne Lebbad

**Affiliations:** 1 Department of Cell and Molecular Biology, BMC, Uppsala University, Husargatan 3, Box 596, Uppsala, SE-751 24, Sweden; 2 Department of Diagnostics and Vaccinology, Swedish Institute for Communicable Disease Control, Nobels väg 18, Solna, SE-171 82, Sweden

## Abstract

****Background**:**

Genetic heterogeneity has become a major inconvenience in the genotyping and molecular epidemiology of the intestinal protozoan parasite *Giardia intestinalis,* in particular for the major human infecting genotype, assemblage B. Sequence-based genotyping of assemblage B *Giardia* from patient fecal samples, where one or several of the commonly used genotyping loci (beta-giardin, triosephosphate isomerase and glutamate dehydrogenase) are implemented, is often hampered due to the presence of sequence heterogeneity in the sequencing chromatograms. This can be due to allelic sequence heterozygosity (ASH) and /or co-infections with parasites of different assemblage B sub-genotypes. Thus, two important questions have arisen; i) does ASH occur at the single cell level, and/or ii) do multiple sub-genotype infections commonly occur in patients infected with assemblage B, *G. intestinalis* isolates?

****Results**:**

We used micromanipulation in order to isolate single *Giardia intestinalis*, assemblage B trophozoites (GS isolate) and cysts from human patients. Molecular analysis at the *tpi* loci of trophozoites from the GS lineage indicated that ASH is present at the single cell level. Analyses of assemblage B *Giardia* cysts from clinical samples at the *bg* and *tpi* loci also indicated ASH at the single cell level. Additionally, alignment of sequence data from several different cysts that originated from the same patient yielded different sequence patterns, thus suggesting the presence of multiple sub-assemblage infections in congruence with ASH within the same patient.

****Conclusions**:**

Our results conclusively show that ASH does occur at the single cell level in assemblage B *Giardia*. Furthermore, sequence heterogeneity generated during sequence-based genotyping of assemblage B isolates may possess the complexity of single cell ASH in concurrence with co-infections of different assemblage B sub-genotypes. These findings explain the high abundance of sequence heterogeneity commonly found when performing sequence based genotyping of assemblage B *Giardia*, and illuminates the necessity of developing new *G. intestinalis* genotyping tools.

## **Background**

The globally occurring diarrhea-causing protozoan, *Giardia intestinalis* (syn. *G. lamblia* and *G. duodenalis*), makes up a species complex of eight different genotypes or assemblages, A-H [1], where assemblages A and B can cause disease in humans [2]. Understanding of the epidemiology of the disease caused by *G. intestinalis* (giardiasis) has been hampered due to the genomic complexity of the parasite (cellular ploidy of 4 N-16 N in two nuclei) [3], along with the genetic heterogeneity that is present in assemblage B *Giardia* isolates [4-6]. The most commonly used genotyping loci; beta-giardin, glutamate dehydrogenase and triose- phosphate isomerase (*bg, gdh* and *tpi,* respectively) have low discriminatory power when applied to assemblage A *Giardia*. Assemblage A sub-assemblages may only be discriminated at a few positions, due to a high level of conservation in these genes in assemblage A isolates, however, three different sub-assemblages have been established at the current loci, namely AI, AII and AIII. In assemblage B on the contrary, high variability in the form of mixed base polymorphisms has been observed at these loci, which has impeded proper epidemiological analyses [7-11]. It is not known if this genetic heterogeneity is due to high allelic sequence heterozygosity (ASH) in assemblage B parasites [12] and/or due to common mixed infections with different sub-groups of assemblage B *Giardia*[5]. Within-species diversity has recently gained increased recognition and has been reported in pathogenic bacteria, fungi as well as in other protozoan parasites such as *Plasmodium falciparum*[13-15]. It has been demonstrated that both polyclonal (infection by phylogenetically divergent clones) and monoclonal (infection by members of a single clone that display micro-heterogeneity) diversity exists in patients with single species infections [13]. This phenomenon is commonly seen in patients harboring chronic infections, which is, interestingly a common problem in giardiasis patients [2]. To date no attempts have been made in investigating whether the occurrence of ASH in sequences generated from clinical assemblage B *Giardia* samples, commonly originate from a single isolate or a mosaic of different isolates. Single cell analyses would be required to resolve this issue. However, isolation of single *Giardia* trophozoites from culture or cysts from clinical *Giardia* samples for the purpose of direct comparative sequence analyses without *in vitro* growth has not previously been performed to the best of our knowledge. Previous methods that have been utilized for the purpose of cloning *Giardia* parasites are labor intensive and do not guarantee the establishment of single cells for molecular analyses [16-19].

Micromanipulation with size-specific micro-capillaries allows very sensitive discrimination, where single cells from a diluted fecal sample can be detected against a background, singled out, and transferred to a pure drop of liquid for re-verification of the clonality of the cell before proceeding to downstream analyses. In the malaria research field, micromanipulation has been applied for qualitative isolation of specific cells from a suspension of mixed cell types and mixed phenotypes, i.e. isolation of *P. falciparum* infected red blood cells (iRBCs) from a rosetting cluster for molecular analyses [20] or the isolation of *P. falciparum* iRBCs at a certain stage in the cell cycle, for molecular analyses [21]. In *Giardia* this approach has been used to isolate single cells for further growth *in vitro* and isoenzyme analysis of the cloned population [17]. The aim of our work was to use micromanipulation to efficiently isolate and sequence single *Giardia* assemblage B trophozoites grown *in vitro*, and single cysts isolated from human giardiasis patients, in order to properly verify genetic heterogeneity on the single cell level without growth *in vitro*. This approach can assess whether genetic heterogeneity identified in clinical assemblage B isolates is due to ASH, mixed sub-assemblage infection or a combination of the two.

## **Methods**

### **Cell lines and clinical samples**

*Giardia intestinalis* GS/M (H7), assemblage B, was cultured in TYI-S-33 at optimal growth conditions [12] and seeded twice weekly prior to single cell analysis. Clinical *G. intestinalis* samples were obtained from patients enrolled in an epidemiology study involving more than 200 giardiasis patients at the Karolinska University Hospital, the Department of Communicable Disease Control and Prevention, Stockholm County Council, and the Swedish Institute for Communicable Disease Control [8]. The Regional Ethics Committee of Karolinska Institutet, Stockholm, Sweden, has approved usage of the clinical samples. Crude DNA from all isolates were subject to PCR and subsequent sequencing of the *bgtpi*, and *gdh* loci and samples used in this study were evaluated based on several stringent criteria; 1) samples had to include assemblage B *G. intestinalis* cysts, 2) cyst load in the patient fecal samples had to exceed 100 cysts per 10 μl concentrated fecal suspension, 3) DAPI stained samples had to yield >80% cysts with intact DNA in the nuclei, 4) sequences generated from multi-locus genotyping (MLG) of the samples had to indicate double peaks in the chromatograms at several positions on one or several of the genotyping loci used in the previous study. Three patient samples were finally included in the study, Sweh197 and Sweh212 which both included assemblage B *Giardia*, and Sweh207, which included a mixed assemblage A and B infection. The patients had prior to infection visited Iraq (Sweh197), Brazil (Sweh212), and India (Sweh207) [8].

### **Purification of cysts from fecal samples**

Fresh fecal samples were examined on wet smears using light microscopy, and stored at 4°C prior to extraction of DNA or purification of cysts. FITC labeled CWP (cyst-wall protein) -specific antibodies (Agua-Glo, Waterborne Inc., New Orleans, LA, USA) and counterstaining with DAPI (4′6-diamino-2-phenyl-indole) were utilized to evaluate the level of viable cysts in each crude patient sample. Cysts were purified from fecal material using a density gradient centrifugation as earlier described [5].

### **Isolation of single*Giardia*cysts and trophozoites**

Single, *Giardia* cysts (Sweh197, Sweh 207 and Sweh 212) and trophozoites (GS/M H7) were isolated according to a previously described methodology [20] with slight alterations. In brief, micromanipulation was performed on diluted and purified cysts from patient fecal samples, as well as chilled diluted *Giardia* trophozoites from cell cultures, using the MN-188 (Narishige, Tokyo, Japan) micromanipulator with sterile micropipettes, and an inverted Nikon Diaphot 300 microscope (Nikon, Tokyo, Japan) (Additional file 1). The sterile pipettes were synthesized “in house” using the P-97 pipette puller (Sutter Instruments, Novato, CA, US) and internal diameters varied from 6 μm to 8 μm based on the differences in size and outer membrane rigidity between the *Giardia* trophozoites and cysts. Prior to micromanipulation, all isolates were diluted down to a working concentration of approximately 10–20 cells per 1 μl solution. Picked cells were transferred to a 2 μl drop of 1XPBS (trophozoites) or ddH_2_O (cysts) and viewed under the microscope for additional verification of proper isolation of single cells. Cells were subsequently transferred to PCR tubes using a micropipette.

A total of 44 *Giardia* cysts from patient samples that had previously been labeled with FITC labeled CWP-specific antibodies were picked and placed on a 12 well microscope slide (Thermo Fisher Scientific, Sweden) in order to verify the specificity of the single cell isolation method. Isolated single cysts, were evaluated by trained, independent microscopists using a Nikon Eclipse E400 Fluorescent microscope (Nikon, Tokyo, Japan). In addition, one of the wells of the slides was always used as a negative control, here liquid was transferred from the fecal suspension onto one of the wells on the 12-well slides and analyzed. Such negative controls were also implemented in the PCR based assays.

### **DNA extraction of single*Giardia*cysts and trophozoites**

Two different methods were evaluated for efficient extraction of DNA from single *Giardia* trophozoites in order to establish a sensitive enough method for the purpose of generating sequences from single cells, where ASH can properly be assessed; 1. Snap freezing/thawing of single trophozoites in 1XPBS at −80°C post-isolation. 2. DNA extraction of single trophozoites using DNAreleasy (NIPPON Genetics Europe, No LS02, Düren, Germany). Isolated single *Giardia* trophozoites were deposited in 2 μl drops of 1XPBS, transferred to PCR tubes containing 3 μl DNAreleasy and treated according to the manufacturer’s instructions (both the short and the long protocols provided by the manufacturer were assayed for extraction of DNA from single cysts). Subsequently, PCR reaction mixtures were added to the samples, to a final volume of 25 μl.

### **PCR and sequencing of target genes for comparative analyses**

Nested PCR was performed on DNA from single cells and trophozoites following the same protocols that have been used on DNA from crude isolates, generating a 530 bp amplicon of the *tpi* gene and a 511 bp amplicon of the *bg* gene [22,23]. Also, in order to verify the assemblages in the clinical samples as well as on all single cysts from isolate Sweh207, assemblage A and assemblage B specific PCRs for the *tpi* locus were performed [10,24].

PCR products were verified on 1.5% agarose gels stained with GelRed (Biotium, Hayward, CA, USA), proper amplicons were purified with Exo-SAP IT^TM^ according to the manufacturer’s instructions (GE Healthcare, Uppsala, Sweden) and sequenced bi-directionally using the BIG DYE 3.1 sequencing kit (Applied Biosystems, La Jolla, CA, USA). Sequenced products were analyzed using the AB 9100 sequence reader (Applied Biosystems, La Jolla, CA, USA), and subsequently examined and aligned utilizing the BioEdit software (Ver. 7.0.5.).

### **Sequences**

The sequences generated in this study were submitted to GenBank, with the following accession numbers [GenBank:JN579665-JN579676] (*tpi* sequence set), and [GenBank:JN579677-JN579688] (*bg* sequence set).

## **Results**

### **Isolation of*Giardia*cysts and trophozoites using micromanipulation**

We have assessed the method of micromanipulation, using custom-made micro-capillaries synthesized with an inner diameter small enough to solely encapsulate single *Giardia* trophozoites or cysts, eliminating the risk of possible transfer of multiple cells. The clonality of all picked cells was further verified by microscopy, prior to transfer into the DNA extraction mixture. As a control, FITC-labeled cysts, purified from patient fecal material were transferred to 12-well microscope slides (n_tot_ = 44 cysts) and fixed by desiccation, followed by the addition of mounting buffer to each well individually. The analysis was performed without the addition of cover slips in order to avoid cross contamination between the wells. The slides were analyzed using a fluorescence microscope and single cysts were present in all 44 wells. Also, all negative controls indicated the absence of *Giardia* cysts.

### **Evaluation of different methods for DNA extraction and efficiency of PCR of single*Giardia*cells**

Two different methods were set up and evaluated in their efficiency of generating DNA from single trophozoites (GS/M-H7) that would yield sequences of high enough quality for the discrimination of ASH. PCR products could efficiently be produced using both protocols, however, the generation of sequences with double peaks in the expected positions showed complete efficiency only when applying the DNAreleasy protocol, as indicated in Table 1. Since the DNAreleasy protocol showed to be the most efficient for the extraction of high quality DNA from single trophozoites, it was subsequently also applied to the single cysts. Both the long and the short extraction protocols provided by the manufacturer were assayed. Applying the long extraction protocol yielded a higher number of positive results in subsequent PCR reactions (data not shown).

**Table 1:** **Comparative sequence analysis of single GS/M trophozoites at the*tpi*locus**

**Isolate**	**Material**	**DNAreleasy**	**GenBank acc no**	**Nucleotide position from start of gene**		
				39*	**45**	**264**
**GS/M**	Cloned sequence		EF688030	A	T	G
	Cloned sequence		EF688028	G	C	A
	Crude isolate		FJ560571	R	Y	R
**GS/M**	Crude isolate		N/A	R	Y	R
**GS/M_3**	Single trophozoites	Not used	N/A	G	C	A
**GS/M_5**				G	C	A
**GS/M_7**				G	C	A
**GS/M_8**	Single trophozoite	Not used	N/A	A	T	G
**GS/M_6**	Single trophozoite	Not used	N/A	R	Y	R
**GS/M_71**	Single trophozoites	Used	JN579671	R	Y	R
**GS/M_72**				R	Y	R
**GS/M_73**				R	Y	R
**GS/M_74**				R	Y	R
**GS/M_76**				R	Y	R
**GS/M_77**				R	Y	R
**GS/M_78**				R	Y	R
**GS/M_79**				R	Y	R
**GS/M_80**				R	Y	R

* This nucleotide position is a substitution pattern proposed as a marker for different B sub-assemblages [25].

### **Sequencing of*Giardia*from culture and at the single cell level**

**Figure 1:**
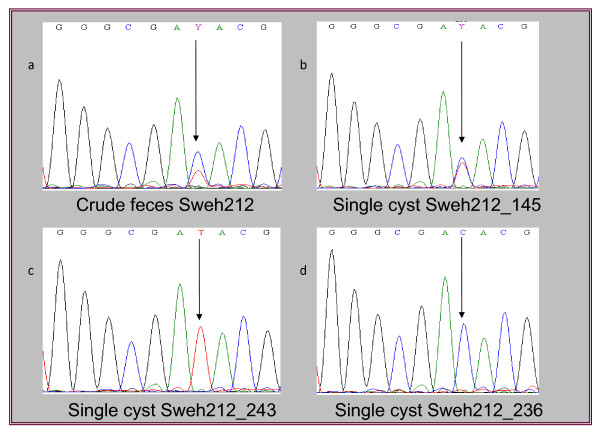
**Sequence chromatograms of nucleotide variations.** Chromatogram of a sequence generated from crude DNA from patient Sweh212, where the position indicated with an arrow shows the presence of a double peak (**a**). Sequencing of single cysts from the same patient indicates the presence of a double peak or ASH at the single cell level (**b**), and importantly, single cyst analyses also show that there are sub-populations present where double peaks do not exist in the same position (**c** and **d**).

Double peaks were stringently validated in the chromatograms of all sequences generated in this study. Only chromatograms with no background bias were used, and positions in the chromatograms where double peaks were clearly visible were represented as ASH in the study. Figure 1, depicts one such position in Sweh212, where double peaks are present in sequences with DNA from crude feces, and single cyst Sweh212_145, but not in single cysts; Sweh212_243 or Sweh212_236 (Figure 1). Sequencing of the *tpi* locus generated from trophozoite cultures of the axenic, assemblage B isolate GS/M-H7, generated double peaks in three positions, namely 39, 45 and 264 (Table 1) with the start codon set as position one. This sequence, along with sequences from public databases [GenBank: EF688030, EF688028 and FJ560571], were used as baseline for the GS/M-H7 analysis in order to define potential polymorphic subgroups when performing the single cell analyses (Table 1). Bi-directional sequencing of single GS/M-H7 trophozoites, with (n = 9) and without (n = 5) the pre-treatment of DNAreleasy, on a 530 bp region of *tpi* was performed in order to verify the occurrence of ASH within single *Giardia* cells. The chromatograms were carefully analyzed with regards to double peaks, and forward and reverse sequences were subsequently aligned. All single GS/M-H7 trophozoites, which were pre-treated with DNAreleasy, displayed distinct double peaks in the same positions as those from the GS/M-H7 crude isolate (Table 1). However, only one (20%) of the single GS/M-H7 trophozoites, that had not been pre-exposed to treatment with DNAreleasy, showed double peaks in all three positions (Table 1). Thus, DNAreleasy increases the amplification efficiency from single parasites.

Bi-directional sequencing was also performed on DNA from clinical single cysts and sequences were aligned using variants of sub-assemblages BIII and BIV, as well as sequences from crude DNA from each respective sample as baselines, where possible. Positions that have earlier been suggested as variable between sub-assemblages BIII and BIV, are highlighted by an asterisk in Tables 1,2,3,4,5[10,25].

**Table 2:** **Comparative sequence analysis of single cysts from Sweh197 at the*tpi*locus**

**Sub-assemblageIsolate**	**Material**	**GenBank acc no**	**Nucleotide position fromstart of gene**			
			39*	**114**	165*	**280**
**BIII/2924**		AY228628	G	C	C	A
**BIV/Ad-19**		AF069560	A	C	T	A
**Sweh197**	Crude stool isolate	JN579672	R	Y	Y	R
**Sweh197_200**	Single cyst	JN579676	R	Y	Y	R
**Sweh197_84**	Single cyst	JN579673	R	C	Y	R
**Sweh197_201**			R	C	Y	R
**Sweh197_86**	Single cyst	JN579674	R	Y	C	R
**Sweh197_98**			R	Y	C	R
**Sweh197_149**			R	Y	C	R
**Sweh197_213**			R	Y	C	R
**Sweh197_196**	Single cyst	JN579675	A	C	C	G
**Sweh197_197**			A	C	C	G
**Sweh197_212**			A	C	C	G
**Sweh197_208**			A	C	C	G
**Sweh197_207**	Single cyst	EU272158	G	T	C	A
**Sweh197_215**	Single cyst	GU56428	G	C	T	A

* These nucleotide positions are substitution patterns proposed as markers for different B sub-assemblages [25].

**Table 3:** **Comparative sequence analysis of single cysts from Sweh212 at the*bg*locus**

**Sub-assemblageIsolate**	**Material**	**GenBank acc no**	**Nucleotide position from start of gene**		
			354*	**369**	**516**
**BIII/ BAH8**		AY072727	C	C	T
**BIV/ Nij5**		AY072725	T	C	T
**Sweh212**	Crude stool isolate	JN579687	T	Y	Y
**Sweh212_143**	Single cyst	JN579688	T	Y	Y
**Sweh212_145**			T	Y	Y
**Sweh212_136**	Single cyst	HM165216	T	T	C
**Sweh212_243**			T	T	C
**Sweh212_236**	Single cyst	HM165214	T	C	T
**Sweh212_242**			T	C	T

* This nucleotide position is a substitution pattern proposed as a marker for different B sub-assemblages [10].

**Table 4:** **Comparative sequence analysis of single cysts from Sweh207 at the*tpi*locus**

**Sub-assemblageIsolate**	**Material**	**GenBank acc no**	**Nucleotide position from start of gene**								
			39*	91*	**162**	165*	168*	**189**	210*	**258**	**423**
**BIII/ 2924**		AY228628	G	C	G	C	C	A	G	C	G
**BIV/Ad-19**		AF069560	A	T	G	T	T	A	A	C	G
**Sweh207**	Crude stool isolate	JN579665	A	C	R	Y	Y	R	R	C	G
**Sweh207_161**	Single cyst	JN579666	A	C	R	Y	Y	R	R	C	G
**Sweh207_227**			A	C	R	Y	Y	R	R	C	G
**Sweh207_222**			A	C	R	Y	Y	R	R	C	G
**Sweh207_166**	Single cyst	JN579667	A	Y	R	Y	Y	A	R	C	R
**Sweh207_228**			A	C	R	Y	Y	R	R	C	G
**Sweh207_220**	Single cyst	JN579669	R	Y	R	Y	Y	A	R	C	R
**Sweh207_224**	Single cyst	JN579670	R	Y	R	Y	Y	A	R	Y	R
**Sweh207_171**	Single cyst	JN579668	A	C	A	C	C	A	G	C	G

*These nucleotide positions are substitution patterns proposed as markers for different B sub-assemblages [25].

**Table 5:** **Comparative sequence analysis of single cysts from Sweh207 at the*bg*locus**

**Sub-assemblage Isolate**	**Material**	**GenBank acc no**	**Nucleotide position from start of gene**						
			**201**	**210**	**228**	**273**	**285**	354*	**537**
**BIII/BAH8**		AY072727	C	C	A	A	T	C	C
**BIV/Nij5**		AY072725	C	T	A	A	T	T	C
**Sweh207_65**	Single cyst	JN579677	C	C	A	A	T	C	T
**Sweh207_66**	Single cyst	HM165209	C	T	A	A	T	C	C
**Sweh207_133**			C	T	A	A	T	C	C
**Sweh207_103**			C	T	A	A	T	C	C
**Sweh207_105**	Single cyst	AY072727	C	C	A	A	T	C	C
**Sweh207_190**	Single cyst	JN579678	C	C	A	G	T	C	C
**Sweh207_61**	Single cyst	JN579679	C	C	A	R	T	C	C
**Sweh207_129**	Single cyst	JN579680	C	T	R	A	T	C	C
**Sweh207_106**	Single cyst	JN579681	C	C	R	A	T	Y	C
**Sweh207_107**	Single cyst	JN579682	C	C	A	A	Y	C	C
**Sweh207_181**			C	C	A	A	Y	C	C
**Sweh207_184**	Single cyst	JN579683	C	Y	A	A	T	C	Y
**Sweh207_186**	Single cyst	JN579684	C	Y	A	R	T	C	C
**Sweh207_183**	Single cyst	JN579685	Y	C	A	R	T	Y	C
**Sweh207_189**	Single cyst	JN579686	Y	Y	R	R	T	Y	C

* This nucleotide position is a substitution pattern proposed as a marker for different B sub-assemblages [10].

Sequencing of *tpi* PCR products from 13 cysts of patient isolate Sweh197 gave rise to six different sequence variants (Table 2). Only one sequence showed the same pattern as the crude isolate with double peaks at positions 39, 114, 165 and 280. The remaining sequences had either overlapping nucleotides at three positions or clean sequences without double peaks at any position (Table 2).

Beta-giardin sequences from six cysts, from sample Sweh212 gave rise to three different sequence variants (Table 3), where one variant indicated the same pattern as that of the crude DNA with double peaks in positions 369 and 516. The other two variants gave rise to sequences without any double peaks; one correlated with sub-assemblage BIV/Nij5 and [GenBank:HM165214] in positions 354, 369 and 516, and the other was identical to [GenBank:HM165216] (Table 3).

Cysts from isolate Sweh207 were investigated at two loci, *bg* and *tpi*. Out of the cysts sequenced at the *tpi* locus, eight were assemblage B and two were assemblage A. This was also verified using assemblage-specific nested PCR primers for *tpi* (data not shown). Sequences from the assemblage A parasites did not indicate any double peaks and corresponded to the sub-assemblage AII reference isolate, JH, [GenBank:U578978]. The eight assemblage B sequences gave rise to five different variants at the *tpi* locus and polymorphisms were present in nine different positions (Table 4). One variant, including sequences from three cysts, was identical to the pattern seen in the crude isolate. Three of the variants had double peaks in two to four positions but lacked double peaks in certain positions compared to the pattern seen in the crude isolate, and one sequence was without double peaks. Sequences generated from crude DNA at the *bg* locus from Sweh207 indicated the presence of both assemblage A and B, therefore no crude DNA sequence is available for comparison at the *bg* locus. However, bidirectional sequencing was performed on 15 single cysts, all of which were of the B assemblage. Comparative analysis of the sequences yielded 11 different variants, and double peaks were present in at least one position in seven of the variants (Table 5).

## **Discussion**

*Giardia* is a unique eukaryote where vegetative trophozoites, as far as we know, harbor two equal, diploid nuclei that contain five different chromosomes each [3]. The two nuclei, in the trophozoite, cycle between a diploid (2 N) and a tetraploid (4 N) genome content in the vegetative cell cycle. During the encystation process the DNA is replicated after cyst-wall formation, giving a cyst with a ploidy of 16 N in four nuclei [3]. The complex genetic makeup of this organism, in combination with published reports of high frequency of sequence polymorphisms in assemblage B *Giardia*[7,8,10,11], has raised the question of whether ASH occurs at the single cell level and how commonly multiple sub-assemblage infections occur in patients. Data from previously published reports have indicated that ASH may occur at the single cell level [6,12]. However, these data are based on sequences generated at the population level from trophozoite cultures, and although there are strong indications of sequence divergence between the alleles, there has still been a necessity of verifying this at the single cell level in order to rule out any bias regarding potential mixed sub-genotypes in the trophozoite cultures. The previously analyzed isolates have been cloned using techniques that do not completely assure the source to be from a clonal cell line, and unintentional mixing of different *in vitro* cultures may cause cross-contamination problems when growing cells in microbiological laboratories. Thus, utilization of the micromanipulation technique rules out the risk of cross contamination since downstream analyses are performed on material from a single cell.

In order to validate allelic sequence divergence at the single cell level of *G. intestinalis*, it is of substantial importance to initiate the PCR reaction with high quality template DNA, where DNA from all alleles is present due to the complex nature of the assay. If the sensitivity of the analysis is not high enough, sequences produced in downstream reactions may indicate false negatives where regions of one or several alleles may not be properly amplified and would thereby not display double peaks in the chromatograms. The implementation of DNAreleasy showed full efficiency in the chromatograms produced from single trophozoites of the GS/M-H7 isolate. A freeze/thaw protocol, which was evaluated simultaneously also produced products in the nested PCR reaction in accordance with Miller et al [19]. This method however, only produced one sequence out of five that allowed the discrimination of double peaks in the chromatograms (Table 1). The *in vitro* establishment of viable assemblage B cysts (GS isolate) is highly inefficient (unpublished data), and therefore impeded the addition of a proper control for the purpose of verifying the presence of ASH in sequences generated from single *Giardia* cysts. DNAreleasy for DNA extraction was the only method sensitive enough to generate sequences where ASH could be detected when analyzing single trophozoites, therefore, two different DNAreleasy protocols provided by the manufacturer were assayed on the clinical cysts. Utilization of the long protocol indicated a higher proficiency in downstream PCR reactions (data not shown).

ASH was seen on the single cell level in all DNAreleasy treated single cells of the GS/M isolate, thus verifying our hypothesis of the occurrence of ASH in single *Giardia* assemblage B parasites. Positions in the sequence on the *tpi* locus, that have earlier been highlighted as variable between different sub-assemblages or haplotypes of GS/M (Table 1) were here verified as double peaks, indicating sequence divergence in the different alleles in single *Giardia* cells. ASH also occurred at the single cell level in the majority of the cysts (21 out of 36 analyzed assemblage B cysts). However, the alignment of all sequences from a single patient sample did not result in the establishment of a consensus sequence. Instead, subgroups formed with unique single cysts or where sequences from two or more single cysts from the same sample origin aligned with complete sequence identity, but where the different subgroups or variants exhibited differences in the sequences in one or several positions. This further strengthens the concept of ASH at the single cell level and it also suggests that all three patients were infected with multiple sub-genotypes of assemblage B *Giardia*. It is noteworthy, that since there are no reference sequences available for any of the cysts isolated from patients in this study it can not be ruled out that some of the sequence variants, where certain sequences do not indicate a mixed base at a position, could be due to a failure in detecting one of the alleles potentially present in a cyst where the DNA may be of sub-optimal quality. Another factor, which could potentially influence misdetection of mixed bases is the possibility that some variant alleles may be present at a lower ratio than others and would thereby not be properly amplified and subsequently detected in the sequencing chromatogram (Tables 2,3,4 and 5). However, in Table 4, positions 39, 91, 258 and 423 indicate the presence of single nucleotides in the sequence from crude stool DNA, but sequences from several single cysts indicate mixed bases at one or several of these positions. Furthermore, many of the sequences from single cysts from the clinical samples indicated ASH at positions that have previously been suggested as variable for sub-assemblages BIII and BIV [10,25]. The common occurrence of ASH in these positions at the single cell level virtually renders these positions inept as discriminatory markers for sub-genotyping of assemblage B *Giardia*.

Sequences generated from single assemblage A and B cysts from patient Sweh207 at the *tpi* locus showed no indication of inter-assemblage recombination on the studied locus. However it would be of great interest to further analyze this on a larger population of samples harboring mixed assemblage A and B infection. The implementation of micromanipulation as an aid in verifying events of genetic exchange in *Giardia* would be highly beneficial. Sequencing based projects of specific target regions where potential recombination events are likely to occur, always include the risk that clinical samples may contain mixed sub-populations. Such bias would however, be completely eliminated if the sequencing was performed on a proficient number of single cysts isolated from populations of cysts from clinical samples using micromanipulation.

*G. intestinalis* has been assumed to be an asexual organism [26], but recent data suggest that this might not be true [27]. Epidemiological and population genetic studies have indicated recombination and allelic exchange between different *Giardia* isolates during infections [28]. Several meiosis-specific genes have been identified in the *Giardia* genome [29]. These genes have shown to be expressed during encystation, at the same time as fusion between the nuclei (diplomixis) has been detected [30]. ASH or single nucleotide polymorphisms have been detected in cultured *Giardia* isolates during genome sequencing projects [12,31,32]. However, considering the fact that the parasite has two diploid nuclei and the level of ASH is surprisingly low: <0.01% in the sequenced assemblage A (WB) and E (P15) isolates and 0.5% in the assemblage B (GS) isolate, it must mean that the parasites can actively reduce the level of ASH and that there must be some kind of communication between the two nuclei, as seen during the diplomixis process [30]. The striking differences in ASH levels between assemblage A and B isolates could imply that the different assemblages have different mechanisms in exchanging genetic material. Another possibility is that assemblage B isolates can fuse in a process similar to the newly discovered sexual process in *Candida albicans* and other pathogenic fungi [14]. In *C. albicans*, two diploid cells fuse and form a tetraploid cell that undergoes parasexual reduction to diploid or often aneuploid cells [14]. Aneuploid *Giardia* trophozoites have been reported [33], which could be remnants of cell fusion and reduction events. Thus, it is possible that the relatively high ASH levels in assemblage B (0.5%) compared to assemblage A (<0.01%) could be due to higher frequencies of cell fusions (sex) in assemblage B isolates. Yet another possibility is that the very low levels of ASH in assemblage A isolates could be due to highly active meiotic components, efficient diplomixis or efficient DNA repair systems.

Recent reports indicate that elevated levels of ASH in the pathogenic fungi, *C. albicans*, are linked to virulence and drug resistance [34,35]. Levert and colleagues have brought light to polymorphisms within bacterial populations and how this may be linked to the generation of virulence phenotypes, such as growth, resistance to stress or resistance to antibiotics [13]. Patient Sweh207, who had a mixed assemblage A and B infection, was subject to treatment failure. Interestingly, after treatment only the assemblage B parasites were present and sequencing indicated high levels of ASH both pre- and post- treatment in the assemblage B portion of the infection [8]. In the same study there were eight other reported cases of suspected treatment failure involving assemblage B infections, where sequencing of the parasites showed double peaks in several positions before and after treatment. Although this has to be further verified, the data brings forth a potential link between elevated levels of ASH and drug resistance in *Giardia*, as is the case in *C. albicans*.

## **Conclusion**

We have developed a methodological pipeline that enables isolation and sequencing analyses of single *G. intestinalis* parasites. The presence of ASH was verified on the single cell level, both in cultured assemblage B trophozoites and in cysts from clinical samples. Also, the analysis of single cysts from patients made it apparent that one clinical, assemblage B containing sample, may often harbor the complexity of ASH in congruence with a mixed sub-assemblage infection. Additional file 2 is a schematic representation of the different possible outcomes in the event of an assemblage B *Giardia* infection. Moreover, the data presented here strongly highlights the necessity of re-evaluating the current molecular epidemiological methods used for sub-genotyping of assemblage B *Giardia*. The concurrence of ASH at the single cell level, and the seemingly high frequency of mixed sub-genotype infections in clinical samples makes it profoundly difficult to verify specific assemblage B sub-genotypes in clinical samples, using the current genotyping tools.

## **Competing interests**

The authors declare that they have no competing interests.

## Author’s contribution

JA and ML carried out the experiments and performed the data analyses. JA, ML and SGS contributed to the design and coordination of the experiments. JA wrote the manuscript. ML and SGS participated in editing the manuscript. All authors have read and approved the manuscript.

## Supplementary Material

Additional file 1 Single *Giardia* cells were isolated by micromanipulation, using micro capillaries with a 6 – 8 μm inner diameter (panel A). Picked cells were transferred to a 2 μl pure drop of 1X PBS for re-verification (panel B), and subsequently transferred to the PCR reaction mixture. Click here for file

Additional file 2 A schematic representation of a mixed infection, where the red and blue bars represent different alleles of the same gene in different *G. intestinalis* sub-assemblages (a), and a single parasite harboring ASH, where red and blue bars indicate different alleles of the same gene within a single cell (b). This is a simplistic, schematic representation of different modes of infection in a giardiasis patient with parasites of different assemblage B sub-assemblages, bringing forth the topics addressed in this study where mixed infection of different sub-assemblages, the occurrence of ASH in a clonal *Giardia* strain, or a mixture of the two may be present in a patient. Thus highlighting an important biological phenomenon in *Giardia*, as well as suggesting a revision of the current strategy used in assemblage B *Giardia* epidemiology. Click here for file
