# Supplementary figures and images for: Allelic sequence heterozygosity in single Giardia parasites
Source: BMC Microbiol. 2012 May 3;12:65. doi: 10.1186/1471-2180-12-65 (PMC3438080; doi:10.1186/1471-2180-12-65)

## Slide 1
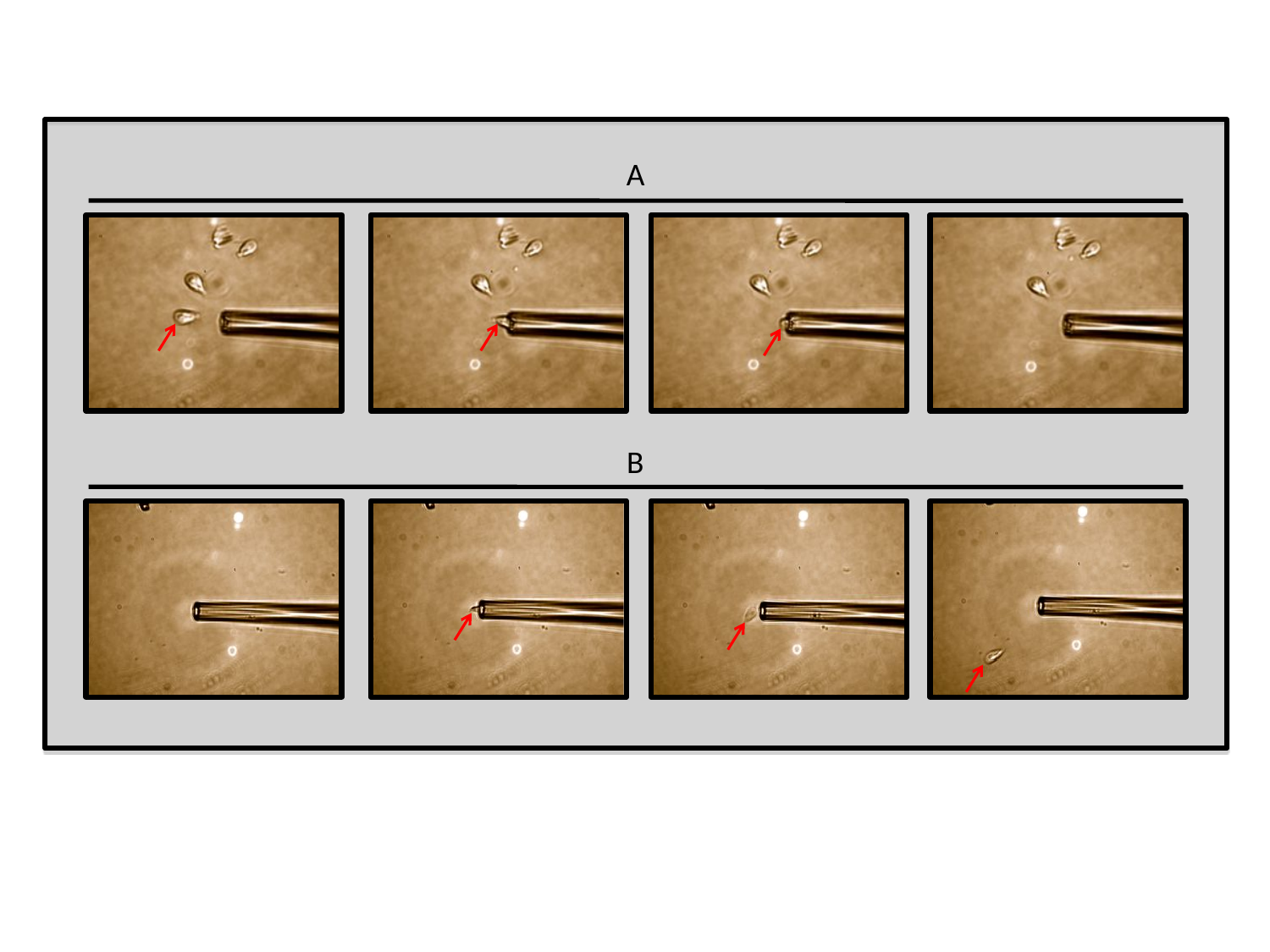

A
B

Supplement: Additional file 1 — Single Giardia cells were isolated by micromanipulation, using micro capillaries with a 6 – 8 μm inner diameter (panel A). Picked cells were transferred to a 2 μl pure drop of 1X PBS for re-verification (panel B), and subsequently transferred to the PCR reaction mixture. [file 1471-2180-12-65-S1.ppt]

## Slide 1
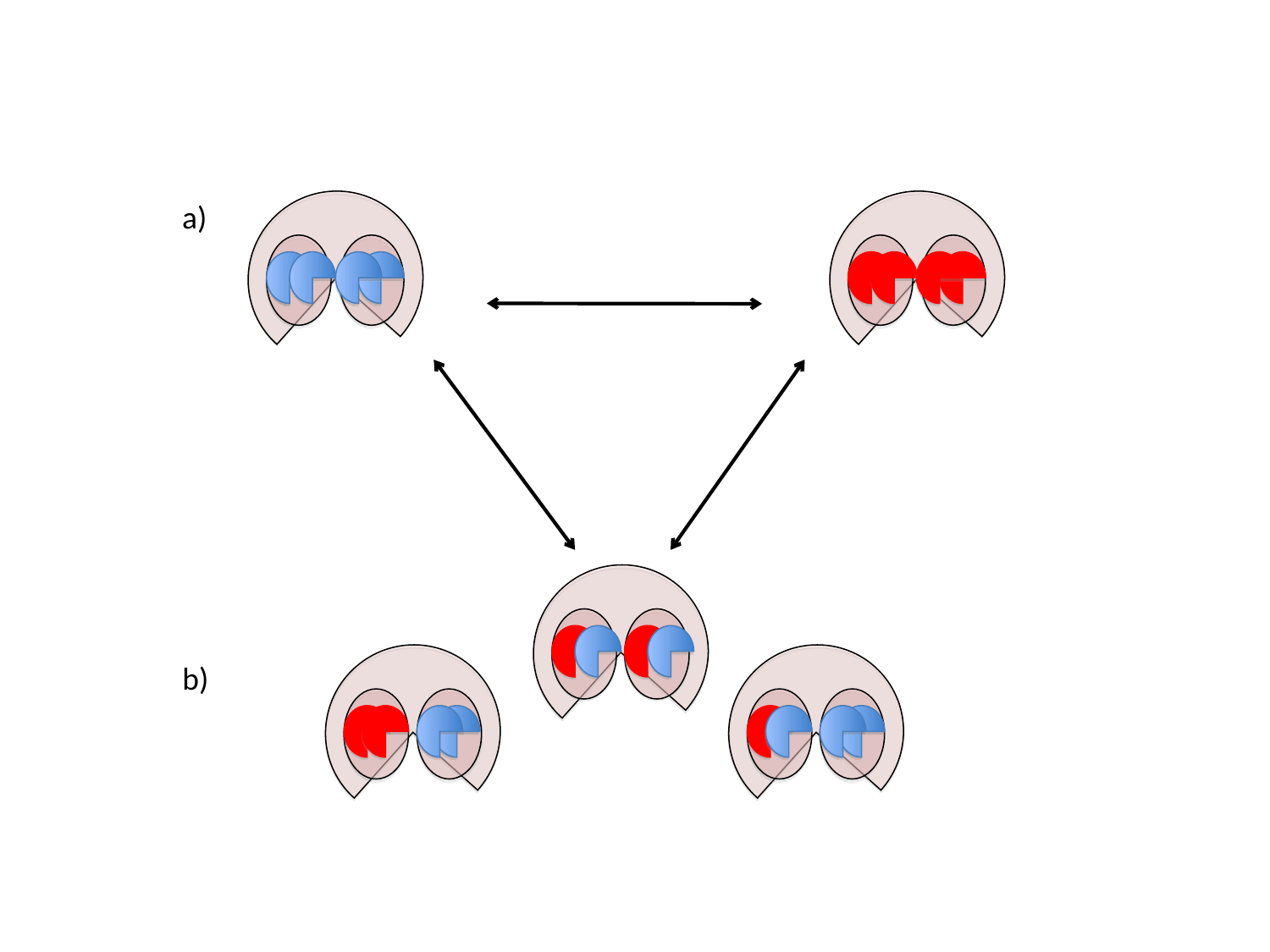

a)
b)

Supplement: Additional file 2 — A schematic representation of a mixed infection, where the red and blue bars represent different alleles of the same gene in different G. intestinalis sub-assemblages (a), and a single parasite harboring ASH, where red and blue bars indicate different alleles of the same gene within a single cell (b). This is a simplistic, schematic representation of different modes of infection in a giardiasis patient with parasites of different assemblage B sub-assemblages, bringing forth the topics addressed in this study where mixed infection of different sub-assemblages, the occurrence of ASH in a clonal Giardia strain, or a mixture of the two may be present in a patient. Thus highlighting an important biological phenomenon in Giardia, as well as suggesting a revision of the current strategy used in assemblage B Giardia epidemiology. [file 1471-2180-12-65-S2.ppt]
